# First-Response ABCDE Management of Status Epilepticus: A Prospective High-Fidelity Simulation Study

**DOI:** 10.3390/jcm11020435

**Published:** 2022-01-15

**Authors:** Paulina S. C. Kliem, Kai Tisljar, Sira M. Baumann, Pascale Grzonka, Gian Marco De Marchis, Stefano Bassetti, Roland Bingisser, Sabina Hunziker, Stephan Marsch, Raoul Sutter

**Affiliations:** 1 Clinic for Intensive Care Medicine, University Hospital Basel, Petersgraben 4, 4031 Basel, Switzerland; paulina.kliem@usb.ch (P.S.C.K.); kai.tisljar@usb.ch (K.T.); siramaria.baumann@usb.ch (S.M.B.); pascalesusanne.grzonka@usb.ch (P.G.); stephan.marsch@usb.ch (S.M.); 2 Department of Anesthesiology and Intensive Care Medicine, Cantonal Hospital of Basel-Landschaft, 4101 Bruderholz, Switzerland; 3 Department of Neurology, University Hospital Basel, Petersgraben 4, 4031 Basel, Switzerland; Gian.demarchis@usb.ch; 4 Medical Faculty, University of Basel, Petersgraben 4, 4031 Basel, Switzerland; stefano.bassetti@usb.ch (S.B.); roland.bingisser@usb.ch (R.B.); sabina.hunziker@usb.ch (S.H.); 5 Department of Emergency Medicine, University Hospital Basel, Petersgraben 4, 4031 Basel, Switzerland; 6 Department of Internal Medicine, University Hospital Basel, Petersgraben 4, 4031 Basel, Switzerland; 7 Department of Psychosomatic Medicine, University Hospital Basel, Petersgraben 4, 4031 Basel, Switzerland

**Keywords:** epilepsy, seizure, convulsive status epilepticus, aspiration, emergency medicine, guidelines, neurology, prospective study

## Abstract

Respiratory infections following status epilepticus (SE) are frequent, and associated with higher mortality, prolonged ICU stay, and higher rates of refractory SE. Lack of airway protection may contribute to respiratory infectious complications. This study investigates the order and frequency of physicians treating a simulated SE following a systematic Airways-Breathing-Circulation-Disability-Exposure (ABCDE) approach, identifies risk factors for non-adherence, and analyzes the compliance of an ABCDE guided approach to SE with current guidelines. We conducted a prospective single-blinded high-fidelity trial at a Swiss academic simulator training center. Physicians of different affiliations were confronted with a simulated SE. Physicians (*n* = 74) recognized SE and performed a median of four of the five ABCDE checks (interquartile range 3–4). Thereof, 5% performed a complete assessment. Airways were checked within the recommended timeframe in 46%, breathing in 66%, circulation in 92%, and disability in 96%. Head-to-toe (exposure) examination was performed in 15%. Airways were protected in a timely manner in 14%, oxygen supplied in 69%, and antiseizure drugs (ASDs) administered in 99%. Participants’ neurologic affiliation was associated with performance of fewer checks (regression coefficient −0.49; *p* = 0.015). We conclude that adherence to the ABCDE approach in a simulated SE was infrequent, but, if followed, resulted in adherence to treatment steps and more frequent protection of airways.

## 1. Introduction

Status epilepticus (SE) is a multisystem disorder. Besides neurologic complications, patients in SE are also at risk for systemic complications, including infections, massive catecholamine releases resulting in neurocardiogenic and pulmonary injury [1,2], and physical injuries due to convulsions or falls [3]. Although current international SE treatment guidelines outline individual treatment steps [4,5,6], they are vague in their indication of sequence, weighting of recommended treatment steps, and lack a more systematic outline, such as the ABCDE approach. According to a systematic review, delayed administration, or wrong dosing of ASDs, seems to be frequent, and is reported in more than 60% of cases [7]. Equally, a recent randomized controlled trial has shown that intubation after established SE is independent of baseline characteristics and early neurologic recovery, though it is strongly associated with site-specific practice pattern variation [8]. Additionally, a recent study showed high variability of SE treatment in EMS algorithms in the US [9]. The systematic priority-based emergency assessment of Airway, Breathing, Circulation, Disability, and Exposure (i.e., ABCDE approach) is the standard examination of critically ill patients as recommended by international organizations [10,11]. It is referred to in several parts of the European Resuscitation Council (ERC) Guidelines 2015 [10], and is a part of the curriculum of the ERC Advanced Life Support (ALS) Provider Course, as well as international standardized courses. SE is a life-threatening neurologic emergency with high morbidity and mortality [12,13,14,15,16], calling for rapid treatment, including airway management, assurance of oxygen and hemodynamic support, protection from physical injuries, metabolic normalization, treatment of underlying pathologies, and administration of antiseizure drugs (ASDs). To what degree such non-compliance with recommended treatment is causally related with suboptimal management and outcome remains to be analyzed, though a connection seems more than likely. Simulator-based studies offer a platform for the design of standardized clinical scenarios that enable detailed investigations regarding clinical practice and effects of the implementation and practicability of treatment guidelines as revealed in a large number of studies mainly dealing with cardiopulmonary resuscitation [17,18,19].

This study aims to:

1. Investigate the frequency and order of correctly performed examination steps of the ABCDE approach by physicians confronted with a simulated scenario of a patient with SE.
2. Further analyze the compliance of SE treatment with the guidelines in relation to performed examinations.
3. Identify risk factors for non-adherence to the ABCDE approach.

## 2. Materials and Methods

### 2.1. Setting and Study Design

This investigator-initiated prospective single-blinded high-fidelity simulator-based study was performed between January 2017 and October 2020, at the simulation center of the medical intensive care units (ICUs) at the University Hospital Basel, a Swiss academic tertiary care center. During the study period, in-house recommendations for SE management were part of the emergency guidelines accessible by all physicians and their use was promoted institution wide. They were in line with current international guidelines [4]. Workshops to train clinical management of a simulated emergency scenario were offered to the medical doctors working as resident physicians in intensive care medicine, emergency medicine, internal medicine, and neurology. Before taking part in the workshop, none of the physicians received prior training regarding the diagnosis and management of patients with SE. The training was offered to all residents of the participating medical specialties during predefined hours of simulator sessions, and during the participants’ regular working hours, without additional payment. Prior to the simulation, all participants were asked to complete a questionnaire that included questions about age, medical knowledge, medical specialization, prior experience with simulator-based training, clinical experience, and hours worked prior to the simulation. To avoid knowledge transfer among participants, all physicians agreed not to share any information about the scenario with their peers, as to not deny them their learning experience. Additionally, the debriefing of the scenario took place after every participant of the day had completed the scenario, and in absence of any other peers. Participants were only aware of other peers participating during the same time slot, and were not informed about the identities of any other peers participating in the study.

The STROBE (STrengthening the Reporting of OBservational studies in Epidemiology) guidelines were followed to enhance the quality of the study [20].

### 2.2. High-Fidelity Simulator Setup

Detailed information regarding the equipment of the high-fidelity simulator center was described in our prior pilot study [21]. In short, and as for prior studies [22], a programmable high-fidelity mannequin (human patient simulator, SimMan^®^, Laerdal Medical AS, Stavanger, Norway) was used to simulate a variety of physiologic and pathologic clinical conditions. The mannequin was able to talk and groan, to present palpable or missing pulses, thoracic excursions and different pulmonary sounds during breathing, blinking and different eye movements, different positions, dimensions and reactivity of the pupils, to display mouth movements, tonic-clonic movements of upper extremities, production of foamy sputum, and enuresis. The breathing frequency, oxygenation levels, heart rate, and non-invasively measured blood pressure were displayed on a bedside monitoring device, as soon as the participating physician chose to install respective monitoring devices, and their values changed in response to the treatment applied. Emergency medications (including vasopressors, antimicrobials, steroids, thiamine, crystalloid fluid and glucose infusions, first- and second-line antiseizure drugs (ASDs), such as various benzodiazepines, valproic acid, phenytoin, levetiracetam, and lacosamide, and third-line ASDs, such as midazolam and propofol, or barbiturates, as bolus or continuous infusion), intubation equipment, suction tubing, flashlights, and dressing materials were available. During the simulation, a nurse (embedded participant) assisted the physician in determining diagnostic results as well as attaching and initiating monitoring devices. The nurse was trained to display a helpful manner, but to act only on commands.

### 2.3. Simulated Clinical SE Scenario and ABCDE Assessment

**Figure 1:**
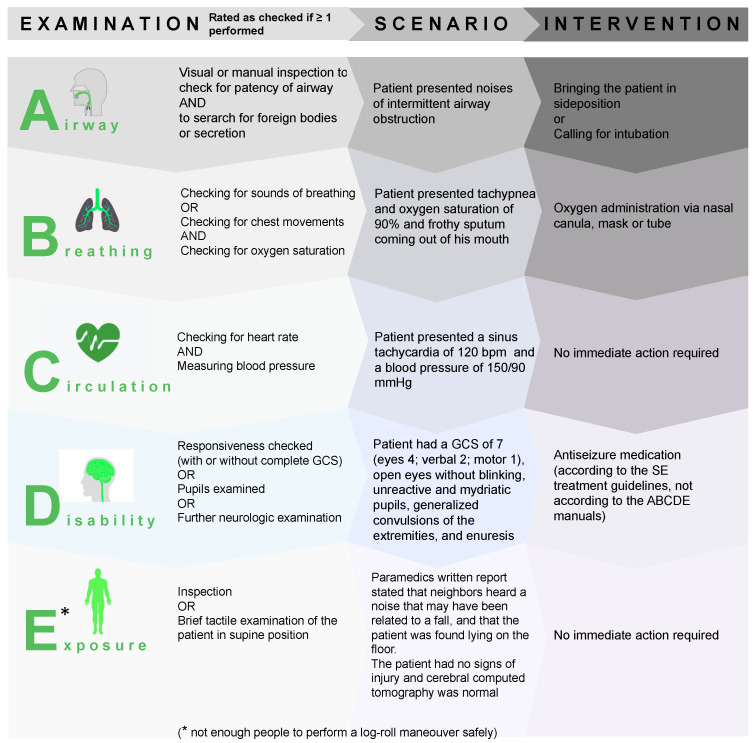
Details of the clinical scenario, the ABCDE examinations, and the measures which should have been taken in response of the clinical findings. GCS = Glasgow Coma Scale.

The simulated scenario of an adult patient in minimal convulsive SE due to alcohol withdrawal was the same as the scenario used in a prior study [21]. Details regarding the clinical scenario, the analyses of the ABCDE approach, and the measures which should have been taken in response of the clinical findings are presented in Figure 1. In short, all participants were blinded to the diagnosis and received a standardized introduction to the simulator room technique prior to simulation. All participants performed the scenario individually, and were informed that they would be acting as the physician on duty in an emergency department, and that a nurse would be available to assist them upon request. The participating physician was called to the emergency department and informed that the patient had minimal convulsions for several minutes, and that he had undergone a cerebral computed tomography, followed by a lumbar puncture due to an unexplained loss of consciousness, with both being unremarkable. The patient showed signs of intermittent airway obstruction by groaning and had foamy sputum in his mouth. If monitored, vital signs showed tachypnea, oxygen saturation levels of 90% with ambient air, a sinus rhythm of 120 bpm, and a blood pressure of 150/90 mmHg. If assessed by the participant, the patient had a GCS of 7. Further clinical details and simulated monitoring data are presented in Figure 1. In the accompanying printed medical records, a written report from the paramedics stated that the patient’s neighbors heard a noise coming from the patients’ apartment that may have been related to a fall, and that the patient was found lying on the floor of his living room. The results of the laboratory workup revealed normoglycemia and lactic acidosis in the blood gas analyses, an unremarkable toxicology screening, a blood count that revealed macrocytosis and elevated liver enzymes, and unremarkable cerebrospinal fluid analyses. The scenario was stopped one minute after the administration of at least one first-line and one second-line ASD. If these drugs were not administered within 20 min, the patient stopped seizing and regained consciousness within another minute to avoid a frustrating experience for the participants. If ASDs were not or insufficiently administered, this was captured independently and had no effect on the recording of compliance with assessing the “D” component of the ABCDE bundle. The maximum duration of the scenario was kept at 21 min.

### 2.4. Outcomes and Measurements

Primary outcomes were defined as correctly performed examination steps “A” to “E”, performance of all examination steps, and examinations performed in the correct order. Secondary outcomes were the performance and time of airway protection (i.e., non-invasive, including head and/or side positioning to avoid aspiration and airway obstruction or loss, or invasive protection, by endotracheal intubation), administration of oxygen, and the administration of first- and second-line ASDs.

### 2.5. Data Assessment

The participants’ performances and the “patient’s” vital signs, as displayed by the monitoring system, were simultaneously video- and audio-recorded using frame-in-frame technology. Data to assess the primary and secondary end points were coded by two independent observers based on the audio- and video-recordings assessed during the simulator training sessions. All actions and utterances were coded second-by-second. Cohen kappa (κ) was used to estimate interrater agreement/disagreement regarding categorical variables. Measurements of continuous variables (i.e., time to action and dosage) were compared. In cases with an interrater disagreement, the videotapes were jointly reviewed until consensus was found. The requirements for being rated as having performed a specific examination of the respective ABCDE system checks is presented in Figure 1.

### 2.6. A Priori Sample Size Calculations

We applied a one-sample proportion test to investigate the null hypothesis that the proportion of participants adhering to SE-guidelines (estimated at 60% based on the results of a systematic review [7]) is equal to the reference proportion of 90% (considered an ideal adherence rate in a clinical setting). Based on these estimates, and assuming two-sided level of significance of 0.05 and power of 0.9, 21 participants are needed to confirm or reject this null-hypothesis. As we investigated adherence in subgroups according to different medical specialties, we aimed to include approximately 20 participants per subgroup.

### 2.7. Statistics

Discrete variables are expressed as counts (percentage) and continuous variables are expressed as medians and interquartile ranges (IQR). Logistic regression was performed to calculate odds ratios (OR) for the associations between participants’ characteristics and deduced SE management. Linear regression models were used to analyze associations between the participants’ characteristics associated with ABCDE execution during SE management. Two-sided *p*-values ≤ 0.05 were considered significant. Statistical analysis was performed with STATA^®^16.1 (Stata Corp., College Station, TX, USA).

### 2.8. Standard Protocol Approvals, Registration, and Consents

Due to the observational design, this study was not registered. The study was approved by the institutional ethics committee and written informed consent was obtained from all participants.

### 2.9. Data Availability Statement

The corresponding author has full access to all data in the study. He takes full responsibility for the integrity of the data, the accuracy of the data analysis and interpretation, and the conduct of the research. The authors have the right to publish any and all data, separate and apart from the guidance of any sponsor.

## 3. Results

### 3.1. Cohort Description

Baseline characteristics of all participants are presented in Table 1. The 74 participants were, on average, 31 (IQR 29–32) years of age, with a median three and a half years of clinical experience, and worked in internal medicine (*n* = 24, 32.4%), neurology (*n* = 19, 25.7%), or in the ICU/emergency room (ER) (*n* = 31, 41.9%), respectively. Males were slightly underrepresented, with the representation being consistent with the current percentage of males in medical education in Switzerland (BAG Statistiken Ärztinnen/Ärzte, 2019). Stated working hours were in agreement with Swiss labor laws (Table 1). Interrater agreement regarding categorical variables was κ = 0.94. SE was recognized by all participants (*n* = 74).

### 3.2. Overall ABCDE Performance

ABCDE performance characteristics are presented in Table 1. Out of the five ABCDE systems, a median of four systems were checked (IQR 3–4). Every participant tested at least one out of five ABCDE systems. A complete check of all systems during the entire simulation was performed by 5% of participants. Airway, breathing, circulation, and disability were all checked at least once (of the two checks within a first and second assessment round as recommended by the World Health Organization) in 96% of all 74 equal scenarios. Exposure (i.e., head-to-toe examination) was examined by 15% of participants.

**Figure 2:**
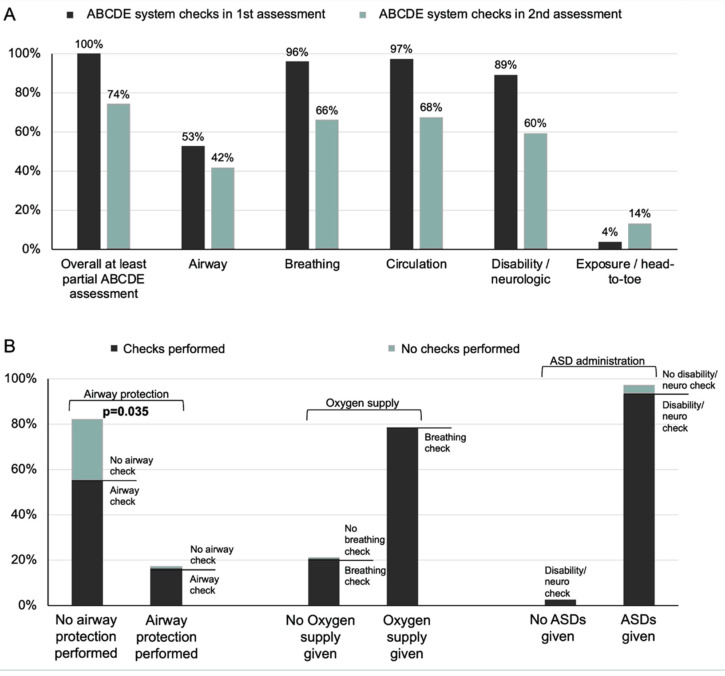
Executed ABCDE system checks (**A**) and deduced treatment measures (**B**) during management of a simulated status epilepticus. ASDs = antiseizure drugs.

The median number of ABCDE systems checked did not vary significantly between primary and secondary assessment (median 3; Table 1). The frequency of ABCDE system checks within the primary and secondary ABCDE assessments are presented in Figure 2A. During the primary assessment participants checked more frequently airway, breathing, circulation, and disability, while exposure (i.e., head-to-toe examination) was performed generally during secondary assessment.

### 3.3. ABCDE Checks, Deduced Management and Adherence to Guidelines

While oxygen and ASD (not being an integral part of the ABCDE approach) were administered independently of breathing checks and neurological examinations, airways were protected significantly more often by participants performing complete airway examination (*p* 0.035; Figure 2B).

Table 2 presents the adherence to the ABCDE system checks and deduced treatment initiations. Disability was checked most frequently, followed by circulation measures, breathing, and airway. Similarly, neurologic treatment (such as the administration of ASD) was performed most frequently, followed by oxygen delivery and measures for airway protection (Table 2).

Details regarding management of SE following ABCDE checks is presented in Table 1. After airway checks, side positionings for airway protection were executed by 17.6% of participants within a median of 2.4 min (i.e., 144 s; IQR 94–234). Of those, 6.7% initiated side positionings within two minutes, and 13.5% within five minutes, as recommended by the treatment guidelines of the Neurocritical Care Society (NCS) and American Epilepsy Society (AES), respectively. A total of 23% of participants called for intubation within a median of 5.2 min (i.e., 310 s). A total of 78.4% of participants supplied oxygen, with 23% administering oxygen within two minutes and 68.9% within five minutes. After disability checks, 79.7% administered first-line ASDs within five minutes, in compliance with the guidelines of the NCS; 95.9% within 10 min, in accordance with the European Federation of Neurological Societies; and almost all participants (98.6%) within 20 min, according to the AES. Second-line antiseizure treatment was administered by 50% of participants within 10 min, compliant with the NCS, and 64.9% within 40 min. The times to administration of ASDs (median 151 s; IQR 123–240 vs. median 162 s; IQR 97–269) and airway protection measures (median 259 s; IQR 247–269 vs. median 239 s; IQR 131–439) were similar between the four participants with, and the 70 participants without, complete ABCDE assessments. 

### 3.4. Participants’ Characteristics and Management of SE

**Figure 3:**
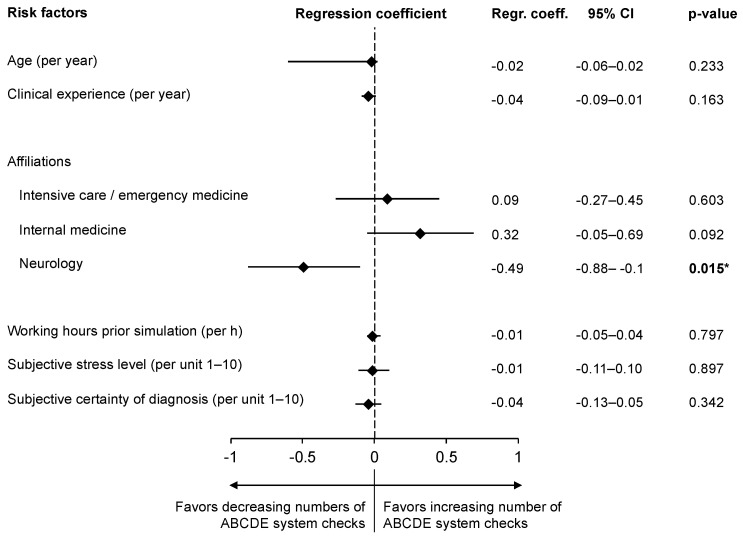
Participants’ characteristics, associated with adherence and non-adherence to the ABCDE approach, during management of a simulated status epilepticus. CI = confidence interval; * Neurologic affiliation was the only significant association with increased odds for not checking airways (OR 0.31, 95% CI 0.1–0.94, *p* = 0.038).

Logistic regression analyses revealed increasing odds for administering oxygen with increasing participants’ age (OR 1.28; 95% CI 1.04–1.57; *p* = 0.019) and with increasing clinical experience (OR 1.74; 95% CI 1.14–2.63; *p* = 0.009). The administration of antiseizure drugs similarly increased with the participant’s age (OR 1.74; 95% CI 1.08–2.83; *p* = 0.024). Conversely, with increasing working hours, the odds for administering oxygen decreased (OR 0.76; 95% CI 0.58–1.00; *p* = 0.05). Effect modification analyses regarding the participants’ characteristics and their effects on the adherence/non-adherence to the ABCDE approach are presented in Figure 3. Participants’ neurologic affiliation was identified as the only significant interaction with decreasing odds for adherence to the ABCDE approach during SE management and additionally poses the only significant relation with increasing odds for not checking airways (OR 0.31, 95% CI 0.1–0.94, *p* = 0.038).

## 4. Discussion

We analyzed the physicians’ emergency response to SE and their approach, compared to the systematic ABCDE approach, in a standardized simulated clinical scenario. The simulated neurologic emergency scenario included an airway problem (i.e., insufficient protective reflexes), a breathing problem (i.e., insufficient oxygen saturation), a disability problem (i.e., status epilepticus) and a possible fall prior to hospitalization (as indicated in the paramedics’ report).

Our analyses reveal that most physicians do not follow the ABCDE approach, and that airway and head-to-toe examination, especially, are performed inconsistently. This reflects the results of our previous study, revealing that the monitoring of vital signs by specialists recognizing SE and being familiar with the SE guidelines is poor [21]. The inconsistent adherence to the ABCDE approach is in line with a previous simulator-based study simulating a critically ill drowned patient, which showed that none of the lifeguards were able to complete the ABCDE approach correctly [23].

Our findings show infrequent attempts to secure and protect the airways. However, physicians examining the airways also proved more likely to protect the airways. Besides neurologic symptoms, patients in SE are also at risk for systemic complications, such as neurocardiogenic and pulmonary injury [1,2], as well as additional respiratory infectious complications [24]. These findings are critical, as failure to protect the airway increases the risk of aspiration and pneumonia, which promotes refractory SE and thereby increases mortality [24,25]. Our results on airway management are further supplemented by a recent randomized controlled trial which shows that intubation after diagnosis of SE is dependent on site-specific practice pattern variation, and independent of baseline characteristics or neurologic recovery [8].

Our study further revealed that participants’ characteristics play a role in their performance of ABCDE derived treatment steps. It found that, with increasing working hours, the frequency of administering oxygen and ASD drops. Although participants’ working hours agreed with Swiss labor laws, further studies are needed to evaluate if scheduled resting periods might increase performance.

Physicians’ age and clinical experience was shown to play a crucial role in the application of oxygen and ASD, the odds increasing with each additional year of professional experience. This likely mirrors the fact that frequent training of standardized basic emergency responses is known to improve outcomes, and has become standard in modern day medicine [26].

Physicians’ affiliation to neurology shows an inverse relation to adhering to the ABCDE approach. This affiliation-related difference in management may be a result of different exposure to patients needing acute management of SE. In other words, emergency medical service teams, in general, are the first group of physicians treating SE, in contrast to neurologists, who usually arrive on the scene after the acute phase management has started. However, treatment protocols outlined by the Neurocritical Care Society and the American Epilepsy Society both describe assessments largely in line with the ABCDE approach [4,5]. Conversely, the European guidelines lack any recommendations concerning a systematic assessment of the patient [6]. Although SE is not an uncommon emergency faced by neurologists [27], to our knowledge there are no large studies on effects of simulator trainings on neurologists’ performances regarding SE. Studies on SE simulation training with ICU fellows report that participants described the scenarios as realistic, improving medical knowledge, heightening comfort in managing neurologic emergencies, and improving leadership skills [28,29,30]. Neurologists, however, were more likely to administer ASDs as compared to internists and intensivists in a prior study [21]. This is important, as delayed treatment and violation of treatment protocols is associated with unfavorable outcomes [31]. While following the ABCDE approach shows no delay in treatment, it may optimize diagnostics and derived treatment measures by its standardized and systematic process, especially improving airway management.

### Limitations

This study has several limitations. First, the single-center design may limit generalizability. However, as most physicians are trained in multiple different medical care centers during their curriculum, our results are likely to be representative for Swiss clinicians. The simulator setting may have limited generalizability and transferability, as it can only reproduce real-life scenarios to a certain degree, especially when it comes to airway assessment, and our results need confirmation in real-life clinical settings. The fact, however, that all participating physicians recognized SE, and that the same simulation has been tested in a pilot study, revealing that SE was reliably recognized, indicates that the simulated scenario was realistic. Moreover, several of our findings are mirrored by clinical studies regarding epileptic seizures and SE, as discussed. In addition, high-fidelity simulation is commonly used as a central training tool for advanced life support, as such simulations provide effective learning opportunities for standardized and controlled clinical practice without putting patients or others at risk [32].

## 5. Conclusions

The results of this standardized observational study regarding the quality of SE emergency response in a high-fidelity simulator show that strict adherence to the ABCDE approach is sporadic, but, if implemented, leads to more frequent protection of airways. Furthermore, physicians’ affiliation to neurology in inversely related to following the ABCDE approach, and is associated with not assessing the airway.

## Figures and Tables

**Table 1:** **Characteristics, clinical assessment, and ABCDE deducted management of participating physicians (*n* = 74).** SE = status epilepticus; IQR = inter quartile range; ASD = antiseizure drug.

**Characteristics of participants**		
Age (years; median, IQR)	31	29–32
Female (*n*, %)	41	55.4
Physicians’ affiliations		
Intensive care/emergency medicine (*n*, %)	31	41.9
Internal medicine (*n*, %)	24	32.4
Neurology (*n*, %)	19	25.7
Years of clinical experience (years; median, IQR)	3.5	2.5–5
Previous simulator training (*n*, %)	34	45.9
Working hours prior to participation (hours; median, IQR)	9	7–10
**ABCDE performance characteristics**		
** ABCDE systems check overall**		
Any ABCDE systems checked (median, IQR)	74	100.0
Number of ABCDE systems checked (median, IQR)	4	3–4
All ABCDE systems completely checked at least once (*n*, %)	4	5.4
Airway checked at least once (*n*, %)	53	71.6
Breathing checked at least once (*n*, %)	73	98.6
Circulation checked at least once (*n*, %)	72	97.3
Disability (neurologic) checked at least once (*n*, %)	71	95.9
Exposure/head-to-toe examination at least once (*n*, %)	11	14.9
** Primary ABCDE system checks**	74	100
Number of ABCDE systems checked (median, IQR)	3	3–4
Order of ABCDE system checks correct (median, IQR)	10	13.5
** Secondary ABCDE system checks**	59	79.7
Number of ABCDE systems checked (median, IQR)	3	2–4
Order of ABCDE system checks correct (median, IQR)	8	10.8
**ABCDE deduced management**		
Deduction from airway check		
Side positioning for airway protection (*n*, %)	13	17.6
Time to side positioning (seconds; median, IQR)	144	94–234
Call for intubation (*n*, %)	17	23.0
Time call for intubation (seconds; median, IQR)	310	241–495
Deduction from breathing check		
Oxygen supply (*n*, %)	58	78.4
Time oxygen supply (seconds; median, IQR)	167	112–219
Deduction from disability (neurologic) check		
ASDs administered (*n*, %)	72	97.3
Number of ASDs administered (median, IQR)	2	1–2
Benzodiazepines administered as first ASD (*n*, %)	72	97.3
Time to first benzodiazepine (seconds; median, IQR)	172	102–270
Times benzodiazepines repeated (median, IQR)	1	1–2
Second-line ASD administered (*n*, %)	48	64.9
Time to second-line ASD (seconds; median, IQR)	471	292–589

**Table 2:** Adherence to guidelines during treatment of patients in status epilepticus. SpO_2_ = peripheral capillary oxygen saturation of the blood; SE = status epilepticus; ASD = antiseizure drug; NA = not applicable due to no or unspecific recommendation. NCS = Neurocritical Care Society, AES = American Epilepsy Society, EFNS = European Federation of Neurological Societies.

	Guidelines of the NCS (8)		Guidelines of the Committee of the AES (9)		Guidelines of the EFNS (10)	
**ABCDE Execution**	***n***	**%**	***n***	**%**	***n***	**%**
Airway check (within 2 min **(8)** or 5 min **(9)**)	25	33.8	34	45.9	NA	
Breathing check (within 2 min **(8)** or 5 min **(9)**)						
Breathing check	34	45.9	49	66.2	NA	
SpO_2_ check	56	75.7	71	95.9	NA	
Circulation check (within 2 min **(8)** or 5 min **(9)**)						
Heart rate check	34	45.9	67	90.5	NA	
Blood pressure check	35	47.3	68	91.9	NA	
Disability (neurologic) check (within 5 min **(8)** or 10 min **(9)**)						
Responsiveness check	71	95.9	71	95.9	NA	
Further neurologic examination	51	68.9	61	82.4	NA	
Exposure/head-to-toe examination	NA		NA		NA	
**ABCDE deduced management**						
Deduction from airway check						
Side positioning for airway protection (within 2 min **(8)** or 5 min **(9)**)	5	6.7	10	13.5	NA	
Deduction from breathing check					NA	
Oxygen supply (within 2 min **(8)** or 5 min **(9)**)	17	23.0	51	68.9	NA	
Deduction from disability check						
Benzodiazepine administration (within 5 min **(8)**, 20 min **(9)**, or 10 min (10))	59	79.7	73	98.6	71	95.9
Second-line ASD administration (within 10 min **(8)** or 40 min **(9)**)	37	50.0	48	64.9	NA	
**Treatment response check** (within 20 min **(8–10)**)	69	93.2	69	93.2	69	93.2

## Data Availability

The corresponding author has full access to all data in the study. He takes full responsibility for the integrity of the data, the accuracy of the data analysis and interpretation, and the conduct of the research. The authors have the right to publish any and all data, separate and apart from the guidance of any sponsor. The data sets supporting the conclusions of this article are available from the corresponding author upon reasonable request, however video files of the participants remain confidential.
